# Supplementary material for: BaRTv2: a highly resolved barley reference transcriptome for accurate transcript‐specific RNA‐seq quantification
Source: Plant J. 2022 Jul 19;111(4):1183–202. doi: 10.1111/tpj.15871 (PMC9546494; doi:10.1111/tpj.15871)
Supplement: Supplementary file 3 — Figure S1. Histogram of end read support to determine window sizes for filtering of Iso‐seq dataset. a) distribution of 5' read ends of transcripts with high coverage (>= 10 reads), b) distribution of 3' read ends of transcripts with high coverage (>= 10 reads). Window sizes used for filtering 5' and 3' ends of transcripts are indicated in C (+/− 10) and D (+/− 30) by vertical lines. Figure S2. Confirmation of predicted TSS by 5' RACE. a) BaRT2v18chr5HG238000. TSS of transcript isoforms are visualised on Integrated Genome Browser (IGB) along with the genomic sequence and the last digits of the co‐ordinates are indicated. 5' RACE products are shown by green arrows. The right hand panel shows the relative abundance (TPM) of expression of isoforms from the different different TSS in inflorescence (INF) and peduncle (PED) tissues. Orange arrow – direction of transcription. b) BaRT2v18chr6HG297070 – the two 5' RACE sites correspond to the most abundant transcripts (legend as in a). c) BaRT2v18chr1HG022010 has a TSS (1552) 250 bp upstream of a second TSS (legend as in a). The 5' RACE signal at 1552 coincides exactly with the major TSS. No signal was detected at 1291 but transcripts using this TSS are much more abundant in other tissues (e.g. roots, rachis, internode – not shown). Figure S3. Histogram of TSS (a) and TES (b) per gene in BaRTv2.18 genes with Iso‐seq support. [file TPJ-111-1183-s003.docx]

**Supplementary Figure 1**

**
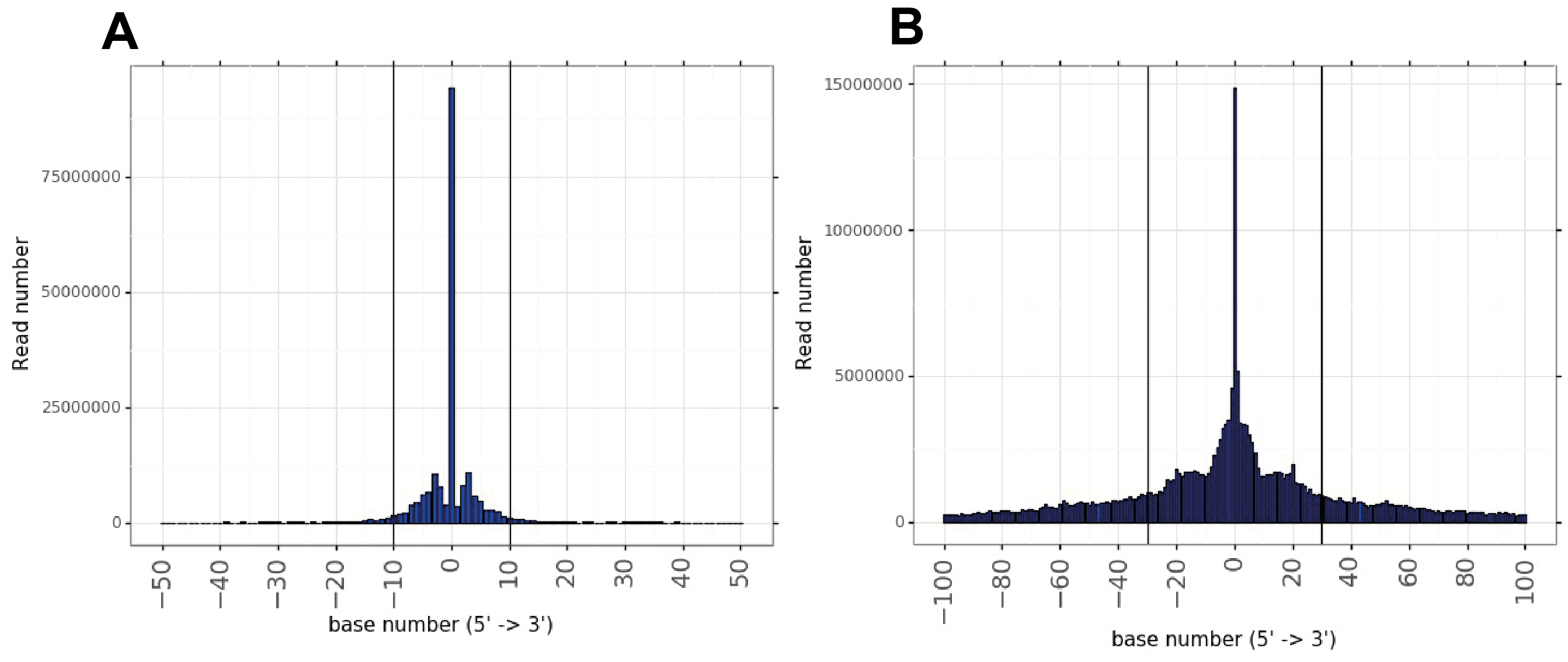
**

**Supplementary Figure 1: Histogram of end read support to determine window sizes for filtering of Iso-seq dataset.** **A** distribution of 5’ read ends of transcripts with high coverage (>= 10 reads), **B** distribution of 3’ read ends of transcripts with high coverage (>= 10 reads). Window sizes used for filtering 5’ and 3’ ends of transcripts are indicated in C (+/- 10) and D (+/- 30) by vertical lines.


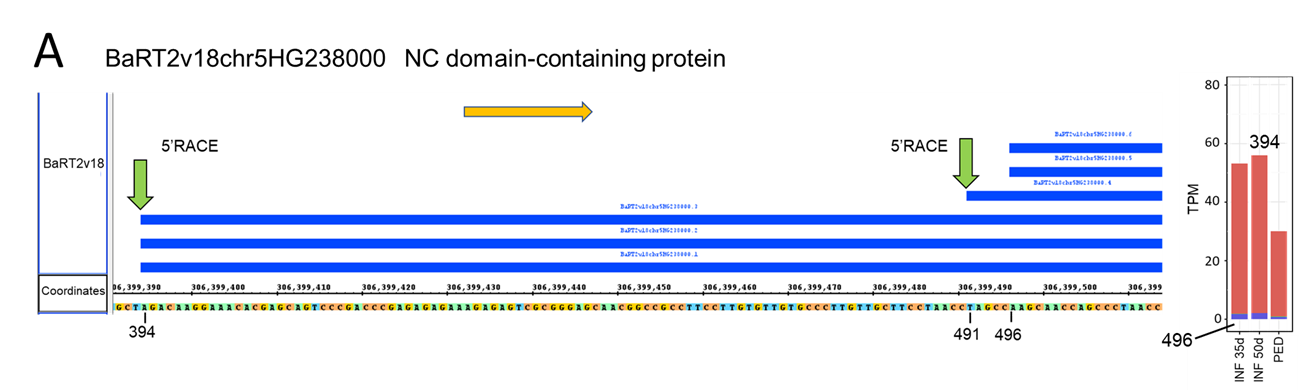


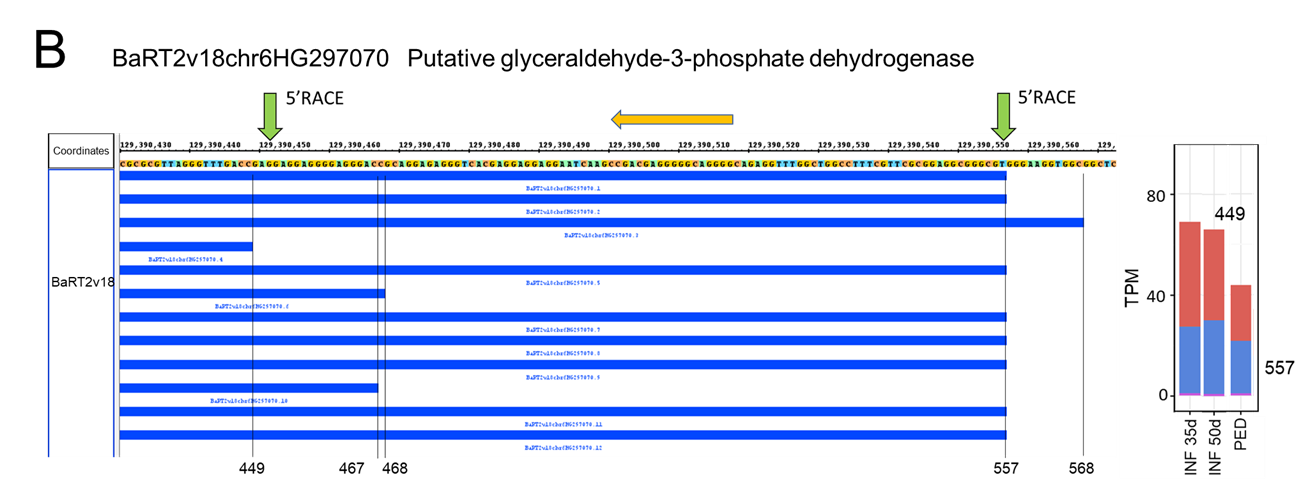


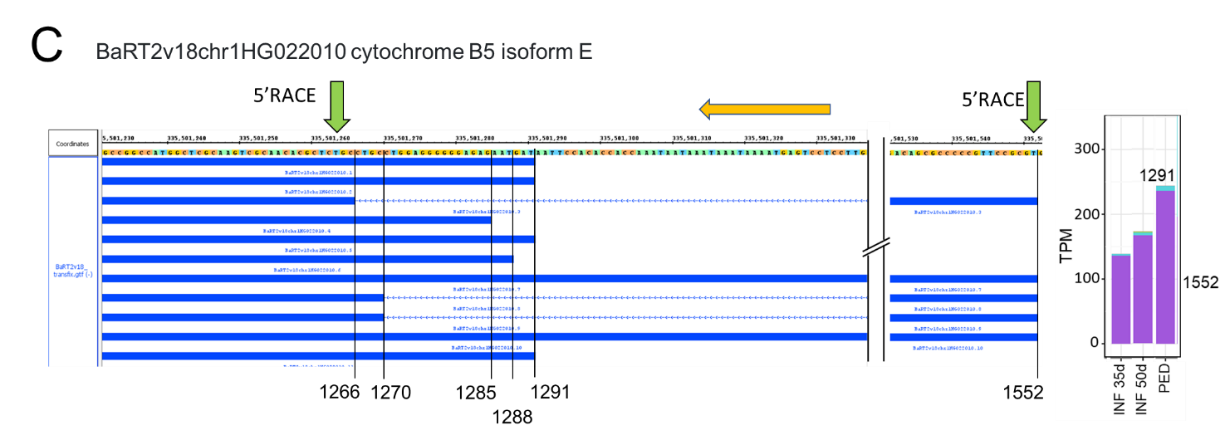


**Supplementary Figure 2. Confirmation of predicted TSS by 5’ RACE.** **A)** BaRT2v18chr5HG238000. TSS of transcript isoforms are visualised on Integrated Genome Browser (IGB) along with the genomic sequence and the last digits of the co-ordinates are indicated. 5’ RACE products are shown by green arrows. The right hand panel shows the relative abundance (TPM) of expression of isoforms from the different different TSS in inflorescence (INF) and peduncle (PED) tissues. Orange arrow – direction of transcription. **B)** BaRT2v18chr6HG297070 – the two 5’ RACE sites correspond to the most abundant transcripts (legend as in A). **C)** BaRT2v18chr1HG022010 has a TSS (1552) 250 bp upstream of a second TSS (legend as in A). The 5’ RACE signal at 1552 coincides exactly with the major TSS. No signal was detected at 1291 but transcripts using this TSS are much more abundant in other tissues (e.g. roots, rachis, internode – not shown).

**
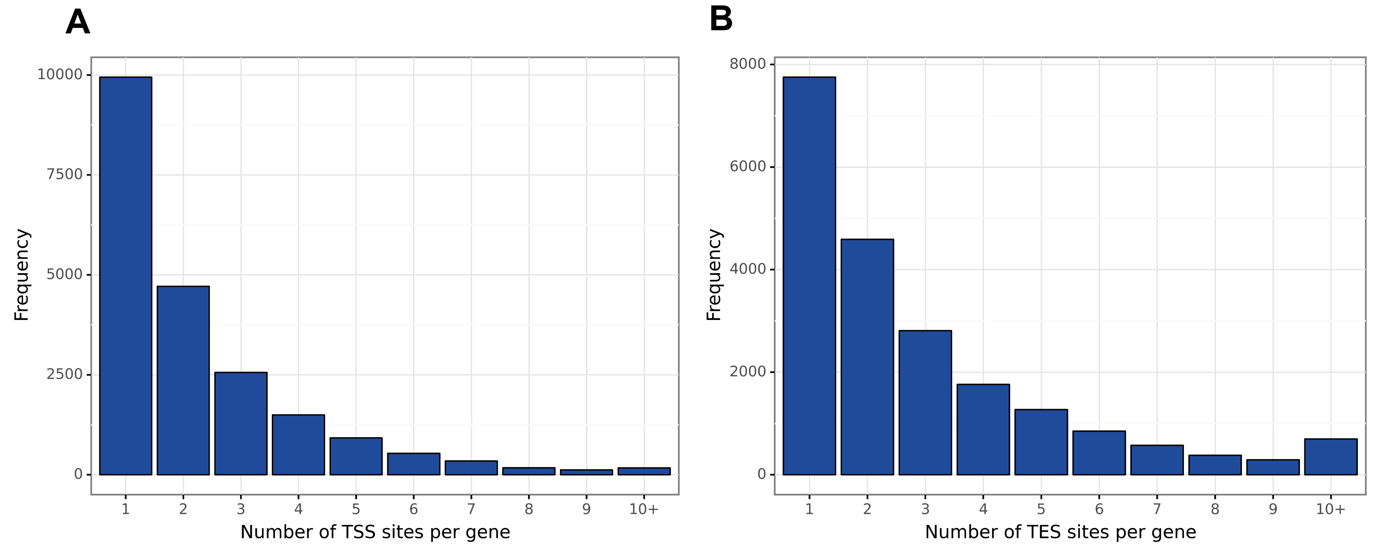
**

**Supplementary Figure 3:** Histogram of TSS (A) and TES (B) per gene in BaRTv2.18 genes with Iso-seq support
